# N-Terminal Cysteine Residues Mediate IL-10 Trafficking and Loading into Extracellular Vesicles

**DOI:** 10.51894/001c.164009

**Published:** 2026-07-31

**Authors:** Lelti Asgedom, Jake Sun, Masamitsu Kanada

**Affiliations:** 1 College of Osteopathic Medicine, Michigan State University

## 10

### Introduction

Extracellular vesicles (EVs) serve as natural carriers for therapeutic biomolecules, including proteins, RNA, and DNA. Our recent studies show that the anti-inflammatory cytokine IL10 can be efficiently loaded into EVs and delivered to proinflammatory macrophages, effectively suppressing their activation. However, the mechanism by which IL10 associates with EV membranes remains unclear. To investigate this, we examined the classically defined signal sequence of IL10 and identified three cysteine residues, hypothesizing that they play a key role in IL10 loading onto EVs. We investigated how IL10 interacts with EVs during cellular release, focusing on whether the N-terminal region is required for EV loading. We analyzed the effects of N-terminal modifications of IL10 and quantitatively assessed IL10 localization in transfected HEK293FT cells. In addition, EVs derived from cells expressing IL10 mutants were isolated, and their IL10 content was evaluated by western blotting.

### Objectives

We hypothesize that post-translational lipidation of N-terminal cysteine residues mediates IL10 association with EV membranes. This study investigates how the N-terminal amino acid sequence of IL10 regulates its interaction with EV surfaces and its incorporation into EVs.

### Methods

IL10 localization and EV association are analyzed using IL10-RFP constructs and fluorescence image analysis. Variants include wild type (WT), N-terminal deletion (dN), and cysteine mutants (C8AC9A, C20A, and C8AC9AC20A) to determine how N-terminus and cysteine modifications affect IL10 trafficking and EV association. To further test the role of cysteine lipidation, cells will be treated with 2-Bromopalmitic acid (2BP), an inhibitor of palmitoylation, to determine whether inhibition of post-translational lipidation affects IL-10 binding to EV membranes.

### Results

Fluorescence microscopy of WT-, dN-, C8AC9A-, C20A-, and C8AC9AC20A-IL10-RFP constructs indicates the N-terminal region is critical for IL10 endosomal localization and EV-associated secretion in transfected HEK293FT cells, suggesting post-translational palmitoylation of N-terminal cysteine residues likely mediates this process.

### Conclusions

These findings highlight a critical role for the N-terminal cysteine residues in IL10 trafficking and EV association, suggesting a previously unrecognized mechanism of cytokine secretion. 2BP-mediated inhibition of palmitoylation will further test whether this modification is required for IL10 loading and release via EVs.

